# Anthropology and ethnobotany in health mediation: an illustration of a clinical, personalized approach for Haitian migrants in a French Guianese hospital

**DOI:** 10.3389/fpubh.2024.1306508

**Published:** 2024-05-27

**Authors:** Marc-Alexandre Tareau, Yoris Demars, Leslie Alcouffe, Ruth Pierre-Louis, Gaelle Walter, Félix Djossou, Nicolas Vignier, Frédégonde About

**Affiliations:** ^1^CIC INSERM 1424, Centre Hospitalier de Cayenne, Cayenne, French Guiana; ^2^Département de Formation et de Recherche - Lettres et Sciences Humaines, Université de Guyane, Cayenne, French Guiana; ^3^Unité de Maladies Infectieuses et Tropicales, Centre Hospitalier de Cayenne, French Guiana; ^4^Ecole Doctorale (ED 587), Université de Guyane, Cayenne, French Guiana; ^5^COREVIH Guyane, Centre hospitalier de Cayenne, Cayenne, French Guiana; ^6^Département de Formation et de Recherche - Santé, Université de Guyane, Cayenne, French Guiana; ^7^Hôpitaux universitaires Paris Seine-Saint-Denis, Hôpital Avicenne and Jean Verdier, AP-HP, Bobigny, France; ^8^IAME, INSERM UMR 1137, Université Sorbonne Paris Nord, UFR SMBH, Paris, France; ^9^Institut Convergences et Migration, Aubervilliers, France; ^10^Faculté de médecine Hyacinthe Bastaraud, Université de Guadeloupe, Pointe-à-Pitre, Guadeloupe

**Keywords:** anthropology, ethnobotany, health mediation, French Guiana, HIV

## Abstract

**Introduction:**

In French Guiana, a European territory in Guiana shield in the Amazon area, close to 40% of the current population was born abroad. In this context, it is important to listen to the experiences of migrants to better understand the difficulties encountered within the healthcare pathways. This is the aim of ANRS Parcours d’Haïti project, an epidemiological, biographical and socio-anthropological study conducted on a representative sample of the Haitian community in French Guiana and focusing on the social determinants of health.

**Methodology:**

Within the framework of this study, the Infectious and Tropical Diseases clinical team of Cayenne Hospital has established close collaboration with health mediators and the ethnobotanist anthropologist of the study. To illustrate the contribution of a personalized approach to health mediation, we report the case of a migrant woman of Haitian origin admitted to the Infectious and Tropical Diseases Unit. We highlight the different socio-cultural aspects addressed and their place in the care process through a thematic discussion and socio-anthropological analysis of the care relationship, based on participatory ethnography and inductive analysis of an in-depth interview with the patient.

**Result:**

This example illustrates the need for a multidisciplinary approach to ensure culturally adapted care for patients. Personal interviews are important because they allow to better take into account the cultural specificities of patients’ experiences and the socio-cultural environment in which they live (and especially, in the case of Haitian patients, their religious affiliation). By allowing them to speak and express themselves freely, they integrate not only their own cultural baggage, but also their own expectations and representations of the disease they suffer from and how it should be treated. Ultimately, this tripartite collaboration between patient, caregiver, and anthropologist or health mediator leads to a better therapeutic alliance.

**Conclusion:**

The analysis of this health care relationship is emblematic of the issue of cultural competence and pre-conceptualizes what intercultural mediation in health care could be, as close as possible to the caregiver and the individual.

## Introduction

French Guiana, a French territory in Amazonia, is one of the largest regions in France in terms of surface area, although its population (around 285,000 in 2020) represents only 0.4% of the French population (1). It is the region with the most dynamic demographic growth in France, excluding Mayotte (+2.5% per year on average between 2014 and 2020), ahead of Réunion (+1.1%) and Île-de-France (+0.8%) (1). French Guiana’s demographic strength is almost entirely due to natural balance. French Guiana is a place with a high population diversity and a very important linguistic and social mix (2). French Guiana is the only European territory in South America with the highest gross national product on the continent (3).

French Guiana is a country of migration. Almost three quarters of the people born and living in French Guiana are the descendants of first- or second-generation migrants, and the population is young (one in two is under 25) and multicultural, with 38% of the registered population of foreign nationality (4). Despite a relatively high overall standard of living, this territory is affected by the precariousness of a large part of its population, particularly migrant communities, with more than 50% of the population living below the poverty line in 2017 and an unemployment rate of 23% in the same year (5). This precariousness particularly affects migrants, with 74% of households living in poverty, compared to 39% for people born in French Guiana. This precariousness is also reflected in access to health care; in 2016, 30.9% of Guianese said they had gone without medical care in the previous 12 months for financial reasons (6).

Migrants may face difficulties in accessing health care, such as language barriers and cultural differences. The health of exiles (immigrants, refugees, asylum seekers or undocumented migrants) is generally better on arrival than that of nationals in host countries but deteriorates rapidly thereafter due to difficulties in accessing care (7, 8).

To better understand the difficulties associated with migration in the health system, we believe it is important to look at the experiences of migrants. This is the aim of the ANRS Parcours d’Haïti project, an epidemiological, biographical and socio-anthropological study conducted by researchers from the Clinical Research Centre Antilles-Guyane at the Cayenne Hospital (INSERM CIC 1424) on a representative sample of the Haitian community in French Guiana, and focusing on the social determinants of health (9). Parcours d’Haïti study is a project that aims to better understand living conditions, access to care and health of Haitian migrants after their arrival in French Guiana. The aim is to enable public health workers to gain a better understanding of these processes, and to develop more relevant prevention initiatives and socio-legal support. It draws on the methods and experience of the ANRS Parcours survey conducted in 2012–2013 in Ile-de-France among people of sub-Saharan African origin (10), while adapting them to the challenges and realities of French Guiana. More than 2,000 people of Haitian origin living in French Guiana were involved in the quantitative and biographical part of the survey throughout the territory. A qualitative component is also carried out by an anthropologist and will shed light on representations of health as well as the differentiated use of biomedical, phytotherapeutic and magico-religious care within the Haitian community. By recruiting Haitians’ patients from healthcare services, this survey brought healthcare teams into contact with the research team, which included an anthropologist and health mediator interviewers. These contacts led to fruitful exchanges and collaborations that went beyond the initial objectives of the research and opened up opportunities for joint work in health mediation.

In this context, the Department of Infectious and Tropical Diseases of Cayenne hospital, which cares for a large number of migrants, has established a close collaboration with health mediation teams and an ethnobotanist anthropologist specialized in Haitian culture. The literature has already shown that mediation in health care can improve care, especially for people in vulnerable situations (11, 12). The aim of this study is to show that an innovative approach based on conversational interviews between a patient and an anthropologist and ethnobotanist is an effective way to improve the therapeutic alliance. We report here on a clinical situation emblematic of this synergy. The case of a migrant woman of Haitian origin hospitalized in the Infectious and Tropical Diseases Department for HIV infection illustrates the contribution of a personalized approach to mediation in health care. First, we’ll report on her hospitalization and the key stages of her care, and then, through a thematic discussion, we’ll highlight the different socio-cultural aspects that were addressed and their place in the care process, before discussing them.

## Methodology

The present work is a case report study enriched by a socio-anthropological analysis of the care relationship, based on participatory ethnography and inductive analysis of an in-depth interview with the patient. These were free interviews, with no predefined interview grid, and the testimonies collected from the patient served as primary data and were manually analyzed thematically.

### Case report

#### “If you tell me i have HIV, i’ll kill myself”

It was in this context that we first met Madame J. on March 2023, a 55-year-old woman born in Haiti who spoke only Haitian Creole and arrived in French Guiana 5 years ago. She was admitted to the nephrology ward for kidney failure and general deterioration, which led to the discovery of her HIV infection. Since her hospitalization, she had become withdrawn, communicating little and eating little. The nephrologists explained to her that she was suffering from renal failure and that her condition required further evaluation in the hospital so that she could receive the appropriate treatment. She was cachectic, withdrawn, and uncommunicative. Once the diagnosis of HIV infection was confirmed, we enlisted the help of an anthropologist and ethnobotanist, an expert in Haitian culture, to prepare the consultation to announce the HIV infection. The announcement came only a few days after she had expressed suspicion and denial about HIV.

On the day of the announcement, unusually, five people gathered in the hospital room: a senior female infectious disease specialist, a junior male infectious disease specialist, a male anthropologist and ethnobotanist, a female health mediator, and the patient. The moment of the announcement was met with apprehension by the caregivers, as the patient had warned that if she was told she was HIV-positive, she would be ready to end her life. However, the announcement went quite smoothly: although she cried when she learned of the diagnosis, the patient did not react too violently, and in fact was quite resigned to the reality of her illness. Significantly, her first question was whether there were any medicinal plants that could cure her disease, to which the infectiologist in charge of the presentation replied that there were none, and that only allopathic treatment could be truly effective in stabilizing her condition. Mrs. J. was then assured by the doctors that she would be well cared for by the hospital’s health teams throughout her treatment. The health mediator and the anthropologist present explained to her in Haitian Creole how her treatment would work and how important it was for her to follow it. Despite the emotional state she was in at the time, she was very attentive and seemed to show understanding and trust.

During her hospitalization in the Infectious Diseases Department for evaluation of HIV infection in the context of an altered general condition, the patient remained withdrawn for a long time. The physicians were limited by the language barrier, and the Haitian-born paramedics described the patient as confused and in refusal of accepting her pathological condition. We then call in a cultural mediator from another organization, the NGO DAAC Guyane (Développement, Animation, Accompagnement, Coopération). This new interview was conducted by a senior male infectiologist and the male Haitian cultural mediator. During this interview, the patient was oriented and spoke clearly about her HIV infection. We also understood that she had been in a situation of oppression since her trip to French Guiana, paid for by her sister: “pay back your trip,” “they offered me to meet men to do it.” She refused. “Sometimes I’ve been offered sexual acts to make a trip, but I prefer to walk.” The person who took her in, her sister, asked her to leave the home. Caregivers wondered if she was even a victim of transactional sex.

Several interviews were then organized between the anthropologist specializing in ethnobotany, who was present on the day of the announcement, the patient, and occasionally the physician. She was more communicative and in a better mood than the rest of the day. She asked questions about the use of certain plants whose medicinal virtues she knew. She expressed full support for biomedical treatments. She asked that the HIV medicine be properly identified. She was also informed that the plants taken were not contraindicated with the prescribed treatment.

She mentioned her desire to leave the hospital as soon as possible, partly because of the food. She also reported that she lacked strength because the hospital meals were low in salt: *“M pa manje manje san sel. Li pa gou, li pa gen pyès fòs annan’l”* (“I do not eat without salt, it has no taste, it has no strength”). In fact, she ate very little, despite the traditional prepared meals brought in by the nursing staff. She did not drink and required intravenous hydration on several occasions.

The patient expressed anxiety about returning home. She wanted to go to a friend’s house, but the friend could not be reached. Allusions to being controlled by the family or to sexual slavery were not found. We did not get a clear picture of the family structure, and her confidants were changeable. Finally, after a visit from one of her relatives who expressed concern for her health and promised to help her with further care, she decided to return to the family home. She was reluctant to tell her family that she had been diagnosed with HIV.

She became more communicative and ate better during her hospital stay. She interacted better with the nursing staff. When she was discharged from the hospital, an organized outpatient follow-up was set up with a city-hospital dedicated health network in French Guiana, the Kikiwi network. To date, the patient has attended all her follow-up appointments. She was gradually regaining her weight.

## Thematic discussion

### Medicinal plants, a strong marker of identity

Herbal medicine occupies an extremely important place among the Haitian population. First, medicinal plants play a clear role as cultural markers in migrant communities, maintained and perpetuated outside their country of origin (13, 14). Even in an urban context characterized by a Western lifestyle, the persistence of herbal medicine practices within the Haitian diasporic community is beyond doubt (15). These cultural practices are deeply rooted in the minds of migrants, and their perpetuation contributes to the alleviation of nostalgia for the homeland. For example, recent studies have shown that Haitians living in French Guiana continue to regularly use a significant number (94 species) of medicinal plants (15). Some of these, such as *Mormordica charantia* and *Ricinus communis*, can even be considered “keystone species” within the community (16).

In this way, the fact of asking about the medicinal herbs used in the fight against the disease is an indication of the strong attachment of the patient toward herbal medicine and her confidence in the medicinal herbs. Naturally, the first exchange between her and the ethnobotanist concerned the pharmacopoeia. Mrs. J. was very surprised to find out that the researcher, who is not Haitian, was very knowledgeable about the medicinal flora of Haiti. She was very interested in discussing the topic with him. As the conversation progressed, she became more and more open to the exchange. The patient even asked him if he knew where to find a plant used to treat certain digestive ailments, *Justicia pectoralis* (*“sèpantyè*” in Haitian Creole), which she had long been looking for. He was able to find the plant, and on a subsequent visit, he brought her a small plant, which further strengthened the bond between them.

### Popular nosography and religious affiliation

Haitian Vodou is a religion that has long been heavily stigmatized (17–19), especially by the Protestant churches whose influence on the Haitian population is growing. This rejection of Vodou in the public arena has driven it underground, and openly asking someone if they are a Vodouist can be a relatively awkward approach. The first thing to do is to ask the person if they go to church, and if the answer is negative, it is generally known that the person is probably a Vodouist (few people in Haiti identify as atheists). In the case of Mrs. J., the anthropologist quickly understood her adherence to Vodou when she confided that she saw spirits (*“lwa”* in Haitian Creole) in her dreams at night. This led to an exchange about Vodou, which, like medicinal plants, was very emancipating for the patient. She seemed liberated to be able to talk freely about these subjects and, above all, to be able to put into words her own concept of illness, linked to her own religious roots.

In fact, during the exchange with the anthropologist, Mrs. J. mentioned her belief that the pathology she was suffering from was probably the result of a witchcraft attack conspired against her by jealous neighbors. She was said to have been “powdered.” This means that malicious people had placed a witchcraft powder on her doorstep or in another place she frequented, with the aim of making her sick. In the specific context of HIV, Haitian Creole speaks of *“vwéyé yon mò sida,”* literally: “sending [the soul of] someone who has died of AIDS.” These cultural representations of HIV, as expressed by the patient, are fully consistent with the holistic cosmovision of Vodou and the explanatory schemas of the disease that they generate. In Haiti, for example, the sudden onset of a serious illness is often associated with an act of sorcery (called in Haitian Creol: “*piaye, dévenn, zen, tjenbwa”*) and is therefore called “*maladi Dyab”* (“Devil’s disease”) or *“maladi vwéyé”* (“sent disease”). This contrasts with “*maladi Bondyé”* (“God’s disease”) or *“maladi natirel”* (“natural disease”), which have a non-sorcerous cause. This fundamental distinction between these two classes of illness is one of the foundations of the nosographic classification specific to Haitian popular medicine and of the dual therapeutic scheme that follows: when a pathology is considered by the patient to have a natural cause, he or she generally favors conventional medical care, whereas when the cause is perceived as supernatural, recourse to traditional treatments is favored, ultimately leading to situations of breakdown in care or even loss of sight, often deplored by caregivers. Mrs. J. herself sums up this view and her own “faith” in Vodou medicine: *“Si m te pe ale Ayiti, m ta fè yo tire maladi saa”* (“If I had gone to Haiti, I would have treated myself”). Thus, while in the case of strictly herbal medicines, there is no real division with conventional medicine, the belief in the magical origin of illness can have real consequences on biomedical treatment, with risks of breakdown and relapse to be taken very seriously.

Obviously, these representations must be heard and taken into account in care for several important reasons. From the patient’s point of view, they represent a subjective reality of the world that must be respected and not disregarded. But they also represent a logical explanation for what is happening to them and will therefore play an important role in their own psychological coping and acceptance of the illness. Moreover, allowing patients to freely verbalize their own cultural interpretation of the pathos they suffer also has the advantage of strengthening the bond of trust between caregivers and patients (who then feel less stigmatized) and, ultimately, of promoting a better therapeutic alliance by emphasizing the vital importance of good compliance, even in the context of mixed care regimens that are very common in multicultural social contexts (20).

### Food

The anthropologist soon began discussing Mrs. J.’s own expectations and representations of food with her, given her refusal to eat at the beginning of her hospitalization. In fact, this situation contributed to her loss of weight and her health to deteriorate. A first point quickly emerged from these discussions: according to Mrs. J., the low salt content of hospital food was a sign of her nutritional weakness. In fact, in popular Creole representations, which are characterized by a humorous view of food (21), salt is considered a very “hot” element that “gives blood” and therefore strength. Apart from the taste, Mrs. J. thinks that eating low-salt dishes would weaken her more than the opposite.

In addition, another point quickly emerged from this exchange: the importance of edible leaves (rarely found in hospital diets) during convalescence in Haiti. Mrs. J. insisted that the first thing she would eat when she returned home would be rice cooked with “leaves” (*“fey”*), considered a very nourishing dish. It should be noted that the generic term “leaves” actually covers a variety of cultivated and wild species that are very popular in Haitian cuisine, one of the most popular of which is *Chamissoa altissima*, *“lyann pannyé”* in Haitian Creole, which is widely eaten by Haitians in French Guiana. This species is sometimes cultivated in home gardens, but it is also harvested as it grows spontaneously in the wild. Other culinary elements are also highly recommended during convalescence, as they are considered particularly restorative, such as vegetable flours (banana, breadfruit, corn and cassava), vegetable juices (beet, carrot, breadfruit, cassava or tomato) and even certain leaves (including *Chamissoa altissima*). In fact, just like medicinal plants, certain Haitian dishes (*“legim ayisyen, tonm tonm, lalo*”) can be considered real identity markers within the community and must therefore be taken very seriously in the care process.

### Difficulties of interpretation

The multicultural nature of the health care teams in French Guiana means that we often have at least one staff member who speaks the patient’s native language. However, our daily experience tells us that even without a complete language barrier, interaction with health care teams remains difficult in several ways. First, the lack of time to engage in these non-medical exchanges and the sometimes-insufficient cultural background are still major obstacles for caregivers. For example, a lack of understanding of Mrs. J.’s family structure led to the initial suspicion that she was part of a prostitution network. This is mainly due to our knowledge of immigrants living with HIV in French Guiana, who are at high risk of contracting the infection during the first year of arrival and some of whom engage in transactional sex (22, 23).In the case of Mrs. J. no evidence was found to support this. The care team often has its own ethnocentric cultural frame of reference, which, in the absence of anthropological hindsight, can lead to ambiguous interpretations of what the patient says. Moreover, the cultural mix inherent in French Guiana’s demographic context produces protean identity profiles (especially among 2^nd^ generation migrants) that are often very complex. The whole exercise of ethnographic interviewing then boils down to carrying out a veritable work of deconstruction, then analytical reconstruction, aimed at deciphering the socio-cultural profile of the patient in order to grasp the influence of his or her cultural background and life course on his or her health trajectory. Religious affiliation(s), age and gender, level of health literacy, and the different cultural and linguistic universes to which the patient refers obviously have a direct impact on the patient’s conception of his or her body and illness, and on the choices he or she makes about treatment. An understanding of all these factors, thanks to the intervention of anthropologists and health mediators, whose role is truly to bridge the gap between medical universes that are sometimes totally opposed to each other, is therefore an indispensable basis for accompanying patients correctly and with dignity throughout the care process.

Difficulties encountered in the case of Mrs. J. and contributions of health care mediation are reported in Table 1.

**Table 1:** Difficulties encountered in the case of Mrs. J. and contributions of health care mediation.

	By the caregiver	By the patient	By the mediators
Difficulties encountered	Language barrier Socio-cultural barrier Caregivers’ personal interpretations Lack of dedicated time Withdrawal	Emotional and social isolation Lack of understanding on the part of caregivers Preconceived images of caregivers	Pluri-culturality, interculturality Multiplicity of caregivers
Contributions of health care mediation	Fluid communication Translation Calming the patient Creating a care alliance Involving the patient in her own care A new framework for discussion with dedicated time for reflection, with a third party in the care process	More expressive More smiling More active in own care Increased confidence in the care system Dedicated time for discussion An open door to be yourself (plants, beliefs)	Multiculturality, interculturality Multiplicity of stakeholders Adaptation to level of understanding The contribution of modesty (intrusive questions from caregivers)

## Conclusion

The need for a multidisciplinary approach to best support patients in their care process is illustrated by this example of an original collaboration between an infectious disease unit, health mediators and an anthropologist. These personal interviews are extremely important because they allow us to better take into account the cultural (and, of course, individual) specificities related to patients’ experiences and their socio-cultural environment (and, in particular, in the case of Haitian patients, their religious affiliation). By giving them a voice and letting them express themselves freely, these interviews make it possible to consider not only their own cultural baggage, but also their own expectations and representations regarding the disease they suffer from and how to deal with it. Ultimately, a better therapeutic alliance is achieved through this tripartite collaboration between patient, caregiver, and anthropologist.

Of course, to be successful, this dialog must take place from a neutral position, with the assurance of a empathic, non-stigmatizing ear. Patients need to feel that they can confide in someone who will take the time to listen to them and respect their views. This freedom of speech helps to establish a bond of trust, which is essential if patients are to be more compliant with hospital care. Through the act of free speech offered to them, patients participate in their own care and become fully engaged in their own health.

Anthropologists play an important role in this type of approach, as their experience in the field enables them to establish a dialog that caregivers would not easily be able to do due to lack of time and/or in-depth knowledge of the cultural practices of these populations, and sometimes a critical and condescending view of patients’ traditional views and practices. Moreover, caregivers often have a burden of stereotypes of their own that can have a distorting effect on the dialog (24). Like a multifaceted facilitator, the anthropologist must learn to adapt to a variety of cultural and individual profiles (age, religion, and so forth), juggling between the different languages spoken, mastering the techniques of discursive iteration, and above all, always maintaining that “axiological neutrality” (25) and attentive, sympathetic listening that are some of the strong epistemological foundations of the anthropological discipline. As this example shows, the anthropologist must be able to bring out enlightening elements for the patient’s care process in a wide variety of areas, such as food, religiosity, and traditional medicine.

This original experience of involving health mediators and a health anthropologist highlights the need for more time and tools for mediation in multicultural care contexts. This study contributes to the existing literature on the benefits of mediation in the health care (12), especially through the anthropology perspective. This can enrich the current discussion on the contours of this multifaceted and interstitial profession (26). The example of Mrs. J. illustrates the need for a more systematic approach to outreach, with individualized, non-judgmental time for conversation that leaves the patient feeling comfortable, heard, and taken seriously. It’s an approach that strengthens the therapeutic alliance.

We believe it is essential to strengthen our support for cultural mediation, taking into account the anthropological and ethnobotanical approach. This study and the forthcoming results of the Parcours d’Haiti survey will enable us to better target the needs of our care services.

Since the Parcours d’Haiti study, other patients have been able to benefit from this approach. These include patients with other diseases (e.g., sickle cell disease) and non-migrants. The response seems to be positive, especially for the therapeutic alliance. The results of the Parcours d’Haiti study will help us to understand the issues involved in treating Haitian migrants. This will allow us to consider systematizing the intervention of a health anthropologist and/or health mediators, especially for complex cases.

## Data availability statement

The original contributions presented in the study are included in the article/supplementary material, further inquiries can be directed to the corresponding author.

## Ethics statement

The ANRS Parcours d’Haïti study was validated by the Protection of Individuals Committee (CPP) Sud-Est I (under number CPP 2021–119 and its substantial modification CPP 2021-119MS02). The patient and all persons mentioned in this article have given their consent to be quoted. Written informed consent was obtained from the individual(s) for the publication of any potentially identifiable images or data included in this article.

## Author contributions

MA-T: Conceptualization, Writing – original draft, Writing – review & editing. YD: Writing – original draft, Writing – review & editing. LA: Writing – review & editing, RP-L: Writing – review & editing. GW: Writing – review & editing. FD: Writing – review & editing. NV: Writing – review & editing. FA: Conceptualization, Methodology, Supervision, Writing – original draft, Writing – review & editing.

## References

[1] Insee. 285 133 habitants en Guyane au 1er janvier 2020 - Insee Flash Guyane - 164. (2022).

[2] Insee. La diversité linguistique marque chaque pan de la culture en Guyane - Insee Analyses Guyane - 54. (2021).

[3] Zéphirin R. Political demography and urban governance in French Guyana: Implications for Latin America and the Caribbean. Singapore: Springer (2020).

[4] Nacher M Rousseau C Bréart G Oueadraogo E Guarmit B Djossou F. Bulletin épidémiologique hebdomadaire (BEH), N°2–3 - Janvier 2020 - Les grands problèmes de santé en Guyane en. (2020): trois exemples de pathologies.

[5] Insee. 29% des Guyanais en situation de grande pauvreté en 2018 - Insee Analyses Guyane - 59. (2022).

[6] Van Melle A Cropet C Parriault MC Adriouch L Lamaison H Sasson F. Renouncing care in French Guiana: the national health barometer survey. BMC Health Serv Res. (2019) 19:99. doi: 10.1186/s12913-019-3895-6, PMID: 30728033 PMC6366016

[7] Aïach P Fassin D. The origins and foundations of social inequalities in health. Rev Prat. (2004) 54:2221–7. PMID: 15736531

[8] Aïach P Fassin D. Social inequalities in health. A dossier to be reopened. Rev Prat. (2004) 54:2219–20. PMID: 15736530

[9] PARCOURS. D’HAÏTI - Institut Convergences Migrations. (2022). Available at: https://www.icmigrations.cnrs.fr/recherche/les-projets/parcours-dhaiti/

[10] Vignier N Desgrees du Lou A Pannetier J Ravalihasy A Gosselin A Lert F. Social and structural factors and engagement in HIV care of sub-Saharan African migrants diagnosed with HIV in the Paris region. AIDS Care. (2019) 31:897–907. doi: 10.1080/09540121.2019.1576842, PMID: 30709323

[11] Richard E Vandentorren S Cambon L. Conditions for the success and the feasibility of health mediation for healthcare use by underserved populations: a scoping review. BMJ Open. (2022) 12:e062051. doi: 10.1136/bmjopen-2022-062051, PMID: 36127102 PMC9490640

[12] Naït Salem R Rotily M Apostolidis T Odena S Lamouroux A Chischportich C. Health mediation: an intervention mode for improving emergency department care and support for patients living in precarious conditions. BMC Health Serv Res. (2023) 23:495. doi: 10.1186/s12913-023-09522-4, PMID: 37194100 PMC10186303

[13] van Andel T Carvalheiro LG. Why urban citizens in developing countries use traditional medicines: the case of Suriname. Evid Based Complement Alternat Med. (2013) 2013:687197:1–13. doi: 10.1155/2013/687197 23653663 PMC3638607

[14] Volpato G Godínez D Beyra A Barreto A. Uses of medicinal plants by Haitian immigrants and their descendants in the province of Camagüey, Cuba. J Ethnobiol Ethnomed. (2009) 5:16. doi: 10.1186/1746-4269-5-16, PMID: 19450279 PMC2690575

[15] Tareau MA Cuerrier A Parent AA Dejouhanet L Palisse M Odonne G. Divergence and convergence in traditional plant-based medicinal practices of Haitian migrants in Montreal, Miami and Cayenne. Hum Ecol. (2022) 50:331–46. doi: 10.1007/s10745-022-00314-8

[16] Garibaldi A Turner N. Cultural keystone species: implications for ecological conservation and restoration. Ecol Soc. (2004) 9:1–18. doi: 10.5751/ES-00669-090301

[17] Munier H. L’illégitimité du vodou et les problèmes de sa médiatisation. Images, sons et récits des Afro-Amériques. Editions des archives contemporaines. (2015):218.

[18] Pierre A Minn P Sterlin C Annoual PC Jaimes A Raphaël F. Culture et santé mentale en Haïti: une revue de littérature [Culture and mental health in Haiti: a literature review]. Sante Ment Que. (2010) 35:13–47. doi: 10.7202/044797ar 21076788

[19] Auguste E Rasmussen A. Vodou’s role in Haitian mental health. Glob Ment Health (Camb). (2019) 6:e25. doi: 10.1017/gmh.2019.23, PMID: 31807309 PMC6880247

[20] Tareau MA. Les pharmacopées métissées de Guyane: ethnobotanique d’une phytothérapie en mouvement [These de doctorat]. Guyane; (2019).

[21] La PA. pocaution cé manman félicité. Communications. (1979) 31:130–44. doi: 10.3406/comm.1979.1474

[22] Nacher M Lucarelli A Huber F Rabier S Douine M Adenis A. HIV-infection among immigrants in French Guiana: high risk during the first years after arrival (2022) 14:27–30. doi: 10.34632/cadernosdesaude.2022.11173

[23] Eubanks A Parriault MC Van Melle A Basurko C Adriouch L Cropet C. Factors associated with sexual risk taking behavior by precarious urban migrants in French Guiana. BMC Int Health Hum Rights. (2018) 18:24. doi: 10.1186/s12914-018-0164-4, PMID: 29884188 PMC5994113

[24] Jan O. L’accueil des indésirables à l’hôpital. VST - Vie sociale et traitements. (2012) 116:107–11. doi: 10.3917/vst.116.0107

[25] Beitone A Martin-Baillon A. La neutralité axiologique dans les sciences sociales. Une exigence incontournable et incomprise. (2016)

[26] Gerbier-Aublanc M. La médiation en santé: contours et enjeux d’un métier interstitiel. L’exemple des immigrant·e·s vivant avec le VIH en France. (2020). Available at: https://www.ceped.org/fr/publications-ressources/working-papers-du-ceped/wp45

